# The value of anti-epileptic therapy as a prophylactic factor for seizures in the management of moderate traumatic brain injury

**DOI:** 10.2144/fsoa-2020-0080

**Published:** 2020-08-28

**Authors:** Konstantinos Faropoulos, Demosthenes Makris, George Fotakopoulos

**Affiliations:** 1 Department of Neurosurgery, Nicosia General Hospital, Nicosia, Cyprus; 2 Department of Head of Critical Care, University Hospital of Larissa, Larissa, Greece; 3 Department of Neurosurgery, University Hospital of Thessaly, University Hospital of Larissa, Larissa, Greece

**Keywords:** anti-epileptic drugs, brain trauma, early seizures, electroencephalogram, late seizures, levetiracetam, moderate traumatic brain injury, post-traumatic seizures, prophylactic anti-epileptic therapy, single post-traumatic seizures

## Abstract

**Aim:**

The value of anti-epileptic therapy in the prophylaxis of post-traumatic seizures.

**Patients & methods:**

All patients received a standard anti-epileptic drug (AED) and were divided into two groups: Group A -with early AED and Group B -with late AED.

**Results:**

Patients (871/1062) met the inclusion criteria. Multivariate analysis demonstrated that computer tomography findings, headache and prior history of brain head injury were independent risk factors of seizures. Only late post-traumatic seizures (LPTS) was significantly associated with AED (p < 0.05).

**Conclusion:**

Early treatment with AED seems to not affect the incidence of lPTS. In addition, an AED with a mean time of initiation of 7.5 days from the moderate traumatic brain injury occurrence could reduce the lPTS incidence.

## Lay abstract

The aim was to study the role of anti-epileptic drugs (AED) as a prophylactic factor for post-traumatic seizures. Patients were divided into two groups: Group A (with early AED). And Group B (with late AED). AED with a mean time of initiation of 7.5 days from the moderate traumatic brain injury occurrence could reduce the late post-traumatic seizure (lPTS) incidence. We believe that an association between the lPTS and AED exists. Both history of brain operation or traumatic brain injury and computer tomography findings like traumatic focal hemorrhagic contusions (temporal), were independent risk factors of lPTS.

Traumatic brain Injury (TBI) is defined as a disturbance in brain function, or other forms of brain pathology, caused by an external force [1,2]. In the USA, there are approximately 30 million injuries per year, which are referred to Emergency Departments, with 16% of these are being referred to as TBIs [3].

Sometimes, this injury is followed by seizures, which could worsen the patient’s outcome and lead to neurologic deterioration or recurrent seizures. Post-traumatic anti-epileptic drugs (AED) are normally utilized to reduce already appearing seizures.

The role of AED in preventing the risk of the development of seizures in people after moderate TBI (mTBI) patients is yet elusive [4]. According to the Brain Trauma Foundation (NY, USA), prophylactic use of anti-epileptic drugs is not advocated as a prophylactic treatment for late post-traumatic seizures (lPTS), even in the case of risk factors [5]. Early post-traumatic seizure (ePTS) does not deteriorate late neurological status [5]. Moreover, anti-epileptic drugs such as levetiracetam are easily used in clinical practice since they do not require blood level counting or loading to reached therapeutic levels.

In this prospective study, we aimed to record the frequency of PTS (ePTS & lPTS) following mTBI, to identify risk factors among clinical and imaging characteristics related to PTS and to determine if prophylactic anti-epileptic drug therapy reduces the risk of PTS. These data could support the potential use of AED after TBI and thus, they might help guide cognitive rehabilitation scheduling [6].

## Patients & methods

### Patients

**Figure 1.**
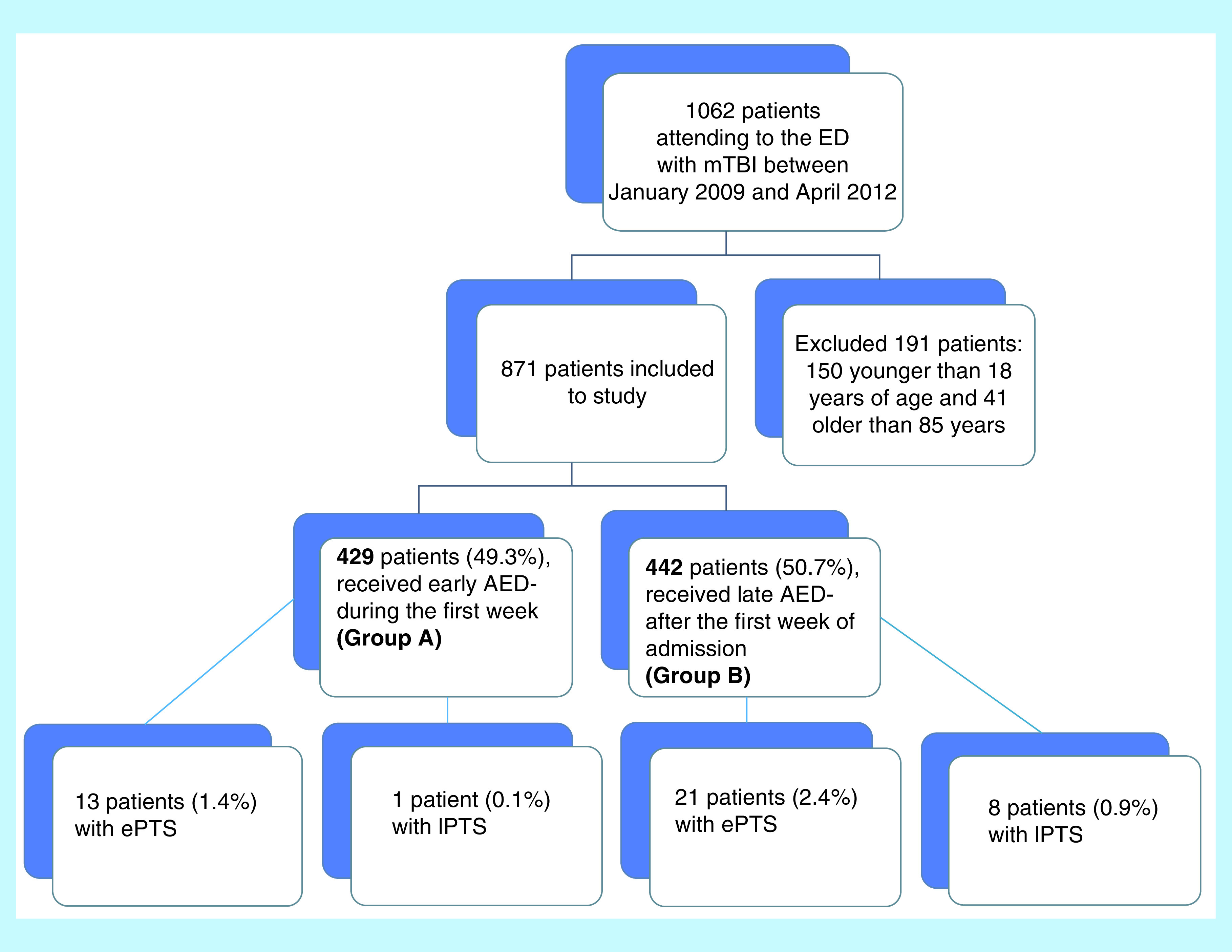
Study selection process. A total number of patients were hospitalized during the study period and were screened for eligibility. AED: Post-traumatic anti-epileptic therapy; ePTS: Early post-traumatic seizure; lPTS: Late post-traumatic seizure; mTBI: Moderate traumatic brain injury.

Patients were recruited from the Emergency Department of the University Hospital of Larisa (Greece), between January 2009 and April 2012 (40 months duration). Exclusion criteria included: less than 18 years of age or greater than 85 years, a medical record of dementia, significant premorbid psychiatric or neurological history and recent use of alcohol or drug abuse (Figure 1). If the patients with mTBI (Glasgow Coma Scale [GCS] 9–12) were eligible for the study, they received a standard AED levetiracetam (1000 mg derived in two doses) daily.

Based on the time when the treating physicians decided to prescribe the AED, patients who entered the study were allocated in two groups; Group A- those who started anti-epileptic therapy at early stages (during the first week of admission) or Group B- those who started at late states (after the first week of admission).

Patients were followed for 5 years in total, having scheduled clinic visits at 1, 2 and 5 years after the incident. Informed consent was obtained.

### Outcomes

The number of patients experiencing seizures after the first week of admission (lPTS) was the primary outcome in this study.

The number of patients experiencing seizures within the first week of admission ePTS was assessed as a secondary outcome.

### Clinical data

Patients were divided into two groups: Group A: with early AED-starting from the very first moment of admission and group B: with late AED – starting 7 days after the incident (a single or/+ multiple seizures). A questionnaire was used to document whether PTS was persistent or and an electroencephalogram was performed to confirm seizures. Moreover, various data were examined as predictor factors, such as the GCS in the moment of admission, chronic smokers, coronary heart disease, medical history of seizures, hypertension, mTBI mechanism (car/motorcycle accident and fall +/or beating), sex, headache age, anticoagulation/antiplatelet treatment, CT scan findings: (traumatic focal hemorrhagic contusions [HCs] in the frontal and temporal lobes or more diffuse punctate HCs, and fractures: convexity or skull base), diabetes, vomiting, stroke incidence, brain surgery or evidence of traumatic brain injury in the past.

CT scan and medical history were utilized for the evaluation of current or/and previous CNS pathology, while alcohol consumption was assessed by the Alcohol Use Disorders Identification Test-Consumption (AUDIT-C) [7] and drug consumption was assessed by the Drug abuse testing using a 24-h timed urine or serum sample [8]. The proportion of people who experienced disability (partially or fully dependent on others in normal activities of daily living) was assessed according to the Karnofsky performance scale index where those patients who were able to carry on normal activity and to work were scored from 80 to 100; those were unable to work but able to live at home and care for most personal needs, were scored from 50 to 70; and patients who were unable to care for self or requires hospital care, were scored from 0 (dead) to 40.

### Statistical analysis

Data are expressed as mean ± standard deviation (SD). Data were assessed for normality using the Shapiro–Wilkes test. Nominal data were analyzed using Fisher’s exact test. Continuous data were analyzed using the Student's t-test or the Mann–Whitney U test as appropriate. Variables significantly associated with univariate analysis were then entered into a multivariable analysis model. A p < 0.05 was considered as statistically significant. Statistical analyses were performed with the use of Statistical Product and Service Solutions (SPSS) software, version 15 (SPSS Inc., IL, USA).

## Results

There were 871 out of 1062 patients (males: 511, 58.6%) who fulfilled study inclusion criteria. From them, 429 patients (49.3%) received early AED (Group A) and 442 (50.7%) received late AED (Group B) (Figure 1). Patients in Group A presented significant differences compared with Group B considering age, GCS on admission, history of brain operation or head injury, headache, traumatic focal HCs in the temporal lobe and skull base fractures (Table 1). In all patients, the electroencephalogram confirmed the seizures. The incidence of seizures (ePTS & lPTS) among the two groups was 3.3% (n = 29) and 1.5% (n = 14), respectively (Table 2). Multivariate analysis (Table 3) demonstrated that a history of brain operation or head injury and computer tomography (CT) findings: traumatic focal HCs in the temporal lobe were independent risk factors of seizures (both ePTS and lPTS). There was a significant difference between patients with ePTS and those with lPTS) only in terms of GCS on admission (p < 0.05) (Table 4).

**Table 1.** Baseline characteristics of patients suffering moderate traumatic brain injury.

Characteristics	All patients n = 871	Group A: early AED, n = 429 (49.3%)	Group B: late AED, n = 442 (50.7%)	p-value
Age, years	32.8 ± 9.7	26.3 ± 8.6	39.0 ± 5.9	**0.001**
Sex (male), n (%)	511, (58.6)	261, (29.9)	250, (28.7)	0.200
GCS of admission, mean ± SD	10.6 ± 1.0	11.1 ± 0.9	10.1 ± 0.9	**0.001**
mTBI Mechanism – Car/motorcycle accident, n (%) – Fall +/or beating, n (%)	553 (63.4) 318 (36.6)	280 (32.1) 149 (17.1)	273 (31.3) 169 (19.4)	0.292 0.230
History of seizures, n (%)	13 (1.4)	8 (0.9)	5 (0.5)	0.372
Hypertension, n (%)	48 (5.5)	23 (2.5)	25 (2.8)	0.849
Coronary heart disease, n (%)	18 (2.0)	8 (0.9)	10 (1.1)	0.680
Chronic smokers, n (%)	163 (18.7)	83 (9.5)	80 (9.1)	0.637
Diabetes, n (%)	21 (2.4)	10 (1.1)	11 (1.2)	0.879
History of brain surgery or head injury, n (%)	8 (0.9)	7 (0.8)	1 (0.1)	**0.030**
History of stroke, n (%)	7 (0.8)	4 (0.4)	3 (0.3)	0.722
Anticoagulation/antiplatelet treatment, n (%)	14 (1.6)	9 (1.0)	5 (0.5)	0.291
Headache, n (%)	89 (10.2)	54 (6.1)	35 (4.0)	**0.023**
Vomiting, n (%)	24 (2.7)	13 (1.4)	11 (1.2)	0.682
RTS, mean ± SD	7.243 ± 0.3	7.342 ± 0.6	7.249 ± 0.7	0.128
CT Findings				
1. Traumatic focal HCs – Frontal, n (%) – Temporal, n (%) – Diffuse punctate HCs, n (%)	111 (12.7) 40 (4.5) 510 (58.5)	48 (5.5) 28 (3.2) 307 (35.2)	63 (7.2) 12 (2.5) 203 (23.3)	0.175 **0.007** 0.187
2. Convexity fractures, n (%)	108 (12.3)	9 (1.0)	99 (11.3)	0.175
3. Skull base fractures, n (%)	102 (11.7)	6 (0.9)	96 (11.2)	**0.001**

Data are presented as mean ± SD, otherwise is indicated.

p-value for the difference between groups was assessed for Nominal data using the Fisher's exact test and for Continuous data with the Mann-Whitney U-test as appropriate.

AED: Anti-epileptic drug; CT: Computer tomography; GCS: Glasgow Coma Scale; HC: Hemorrhagic contusion; PTS: Patients with post-traumatic seizure; RTS: Revised Trauma Score; SD: Standard deviation; TBI: Ttraumatic brain injury.

**Table 2.** Patients’ outcomes.

Outcome	Group A, n = 429 (49.3%)	Group B, n = 442 (50.7%)	p-value
ePTS, n (%)	21 (2.4)	13 (1.4)	0.162
lPTS, n (%)	8 (0.9)	1 (0.1)	**0.019**
Karnofsky performance scale index, n (%)	97.4 ± 9.5	97.7 ± 9.35	0.102

Data are presented as mean ± standard deviation, otherwise is indicated.

ePTS: Early post-traumatic seizure; lPTS: Late post-traumatic seizure.

**Table 3.** Independent risk factors of seizures (lPTS & early post-traumatic seizure) after multivariable analysis.

Name	p-value	OR	CI (95%) Lower–upper
1. Age	0.324	0.030	0.006–0.019
2. GCS of admission	0.312	0.027	0.103–0.323
3. History of brain surgery or head injury	**0.003**	0.083	0.572–2.898
4. Headache	0.742	0.009	0.410–0.292
5. CT findings: traumatic focal HCs (temporal)	**0.000**	0.577	4.995–6.064
6. CT findings: Skull base fractures	0.233	0.035	0.136–0.557

p-value for the difference between groups.

CT: Computer tomography; ePTS: Early post-traumatic seizure; GCS: Glasgow Coma Scale; HC: Hemorrhagic contusion. lPTS: Late post-traumatic seizure; OR: Odd ratio.

**Table 4.** Comparison between patients with early post-traumatic seizure and with lPTS.

n = 43 with seizures	ePTS, n = 34 (79.0%)	lPTS, n = 9 (20.9%)	p-value
Age, years	49.2 ± 18.3	39.5 ± 22.9	0.468
Sex (male), n (%)	10 (23.2)	3 (6.9)	0.820
GCS of admission, mean ± SD	10.5 ± 0.9	9.7 ± 0.9	**0.031**
mTBI Mechanism – Car/motorcycle accident, n (%) – Fall +/or beating, n (%)	21 (48.8) 13 (30.2)	7 (16.2) 2 (4.6)	0.370 0.357
History of seizures, n (%)	1 (2.3)	1 (2.3)	0.301
Hypertension, n (%)	14 (32.2)	3 (6.9)	0.669
Coronary heart disease, n (%)	7 (16.2)	1 (2.3)	0.516
Chronic smokers, n (%)	3 (6.9)	1 (2.3)	0.834
Diabetes, n (%)	9 (20.9)	2 (4.6)	0.795
History of brain surgery or head injury, n (%)	5 (11.6)	1 (2.3)	0.782
History of stroke, n (%)	5 (11.6)	0	–
Anticoagulation/antiplatelet treatment, n (%)	12 (27.9)	2 (4.6)	0.457
Headache, n (%)	6 (13.9)	1 (2.3)	0.637
Vomiting, n (%)	6 (13.9)	2 (4.6)	0.754
RTS, mean ± SD	7.332 ± 0.5	7.334 ± 0.5	0.231
CT Findings			
1. Traumatic focal HCs – Frontal, n (%) – Temporal, n (%) – Diffuse punctate HCs, n (%)	20 (46.5) 8 (18.6) 29 (67.4)	7 (16.2) 7 (16.2) 2 (4.6)	0.294 0.587 0.934
2. Convexity fractures, n (%)	7 (16.2)	2 (4.6)	0.915
3. Skull base fractures, n (%)	5 (11.6)	0	–
**Groups**			
Group A	13 (30.2)	1 (2.3)	
Group B	21 (48.8)	8 (18.6)	0.123

Data are presented as mean ± SD, otherwise, it is indicated.

p-value for the difference between groups was assessed for Nominal data using the Fisher's exact test and for Continuous data with the Mann–Whitney U-test as appropriate.

CT: Computer tomography; ePTS: Early post-traumatic seizure; GCS: Glasgow Coma Scale; HC: Hemorrhagic contusion; lPTS: Late post-traumatic seizure; PTS: Patients with post-traumatic seizure; RTS: Revised Trauma Score; SD: Standard deviation; TBI: Traumatic brain injury.

Clinical outcomes are demonstrated in Table 2. Only lPTS was significantly associated with AED (p < 0.05). Overall, lPTS was 1% (9/871 patients) and it was higher in Group A when compared with Group B.

**Figure 2.**
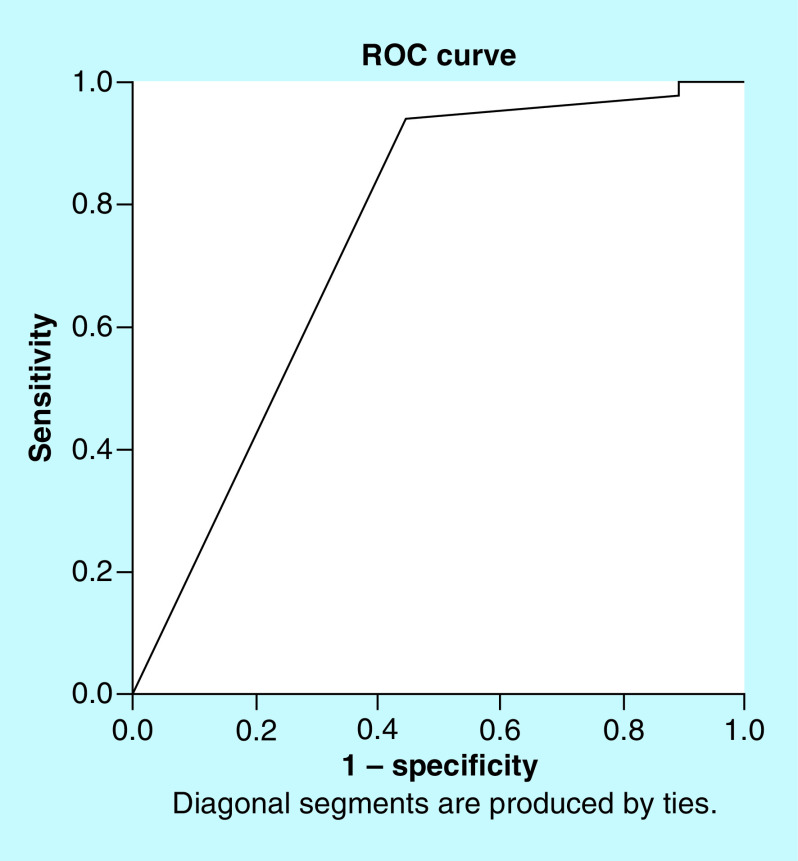
ROC curve is shown with a sensitivity of 95% and specificity of 67% (95% CI: lower–upper [0.545–0.947]; p = 0.01) that an AED with a mean time of initiation of 7.5 days from the mTBI incidence could reduce the lPTS. AED: Post-traumatic anti-epileptic therapy; lPTS: Late post-traumatic seizure; mTBI: Moderate traumatic brain injury; ROC: Receiver operating characteristic.

Receiver operator characteristic (ROC) analysis determined that 7.5 days duration of AED presented significant predictive value for lPTS (sensitivity of 95% and specificity of 67% [95% CI: 0.545–0.947]), p = 0.01 (Table 5 & Figure 2).

**Table 5.** Statistical findings for receiver operator characteristic.

	Area	Std Error	CI (95%) Lower–upper	p-value
ePTS	0.726	0.055	0.619–0.834	0.001
lPTS	0.746	0.102	0,545–0.947	0.011

Data are presented as n (%), otherwise is indicated.

ePTS: Early post-traumatic seizure; lPTS: Late post-traumatic seizure; ROC: Receiver operator characteristic; Std: Standard.

## Discussion

The cardinal findings of the present study that included patients with mTBI were: PTS incidence was 4.8%; lPTS was 1% and ePTS was 3.8%. lPTS was statistically significant with AED, history of brain surgery or head injury and CT findings: traumatic focal HCs (temporal), were independent risk factors of lPTS and AED with a mean time of initiation of 7.5 days from the mTBI incident could reduce the occurrence of lPTS.

According to a study by Wang *et al.* [9], 59.9% of patients were identified as having PTS after TBI. Hence, there is an increased possibility for developing PTS after TBI to patients with severe TBI (patients with low GCS) [10], frontal–temporal lobar contusion and linear skull fracture. In our study, the incidence of PTS in mTBI patients was low 4.8%. Only patients who had previously been operated on or had an injury on the brain and those were diagnosed with temporal lobes focal HCs and skull base fractures during the accident, seemed to develop PTS.

Phenytoin, carbamazepine, sodium valproate and phenobarbitone have been demonstrated to be effective in the treatment of partial or generalized tonic-clonic seizures [11]. Furthermore, Phenytoin is effective in the prevention of ePTS when administered within the first-week post-injury [11]. Notwithstanding, many papers concluded that the use of aforementioned the drugs appears to prevent lPTS [4], regardless of the investigations or the type of injury [11].

On the other hand, some argue that the risk of lPTS occurrence was reduced by AED compared with placebo or usual cure, although the difference was not statistically significant [10]. According to a study by Temkin *et al.* AED, a valuable result by reducing seizures was achieved during the first week after head injure [12]. Our study demonstrated that only the AED with a mean time of initiation of 7.5 days from the mTBI incidence could reduce the lPTS. Instead, an AED at the very first moment from the event had not any effect in reducing seizures.

In many reports, the prophylactic use of anti-epileptic drugs is not recommended for the prevention of ePTS [13]. Additionally, other studies mentioned that ePTS are not associated with worse outcomes [5]. These different results may be due to the comparatively low or variable incidence of PTS in published series [13]. Our study dialed with mTBI patients and showed no definitive significant difference, between AED and ePTS; p = 0.162 (Table 2).

PTS is not generally in attendance of all mTBI cases. Over the last decade, several studies have identified the frequency of AED which accounts for 20% of acquired seizures in the general population [14]. Other studies also have shown unreliable incidence rates [9,15]. The reason for that variability in the occurrence of PTS may be derived by the differences in study design and population, in other words there was no homogeneity in participants, where the pathophysiologic basis for PTS and the expand of the damage may differ considerably. In our study, we included homogenous populations with mTBI and PTS incidence was 4.8% (43/871 patients) – lPTS was 1% (9/871 patients) and ePTS was 3.8% (34/871 patients). lPTS was higher in Group A when compared with Group B (p < 0.05), indicating that lPTS could act as an independent factor of AED. Thus, early therapy – during the first week of admission – with AED seems to not affect the incidence of lPTS. ROC analysis demonstrated a value of 7.5 days presented with a sensitivity of 95% and specificity of 67%. This means that the AED with a mean time of initiation of 7.5 days from the mTBI incidence could reduce the lPTS. On the other hand, in this study the incidence of seizures among the groups was 43 (4.9%) as 29 patients (67.4%) were in Group A (patients with early prophylaxis) – 21 patients (48.8%) developed seizure at an early stage and only eight patients (18.6%) showed late epileptic seizure. This probably could be explained: first, from the fact that these patients mainly had a lower GCS – as they divided into Group A were the physician decided early prophylaxis – hence they had a greater possibility to developed seizure or second, probably in the first 7 days, the drug levels in these patients were influenced by many factors caused by TBI.

In previous studies, PTS has been associated with multiple risk factors [16] such as young age, depression, family history, alcohol and factors of increasing damage harshness such as penetrating injuries [17]. Others have been identified as a risk factor for PTS the distraction of the blood–brain barrier [17]. More recently, it has been suggested that genetic factors [18] and people with a history of depression are at high risk of developing PTS [19]. Another interesting point is that most studies focused on severe TBI only [13], while our attention was concentrated in mTBI. On the other hand, our study showed that young age was significantly different (p = 0.001) among the groups (Table 1), but multivariable analysis demonstrated that age was not an independent risk factor of seizures (p < 0.05; Table 3).

It should be underlined here that some points should be taken into consideration in the explanation of our findings. First, is that a number of patients (6.7 %) who were lost to follow-up. Nevertheless, we believe that this loss was negligent, and were equally distributed in the two groups (3.5 and 3.2 % in Group A and B respectively), thus this population may not have affect our findings. Next, patients over 85 or less than 18-year old were excluded from the study. Older patients were excluded since they frequently present disabilities due to pathological causes or cognitive problems that could obscure TBI related to AED evaluation [20,21]. Young patients (less than 18-year old) also, due to different recovery rates or duration of symptoms when compared with older patients, could disturb the homogeneity in the study population [22–26]. Third, in this protocol, we used only CT studies that may lack sensitivity when damages are small. MRI scan might be more perceptive but was difficult to be applied in such a large scale. Fourth, in our study as we did not perform any measurement for the blood levels of Levetiracetam, perhaps patients with more severe brain injury could have affected Levetiracetam levels by many factors.

Other limitations were that this study uses data from a single-center and may have been underpowered to detect a difference in the primary outcome since the incidence of PTS is very low. A larger cohort could demonstrate a more significant difference in the incidence of early PTS among TBI patients who received prophylaxis and those who did not.

## Conclusion

We believe that an association between the lPTS and AED exists. In our study, ePTS incidence in mTBI cases was approximately 3.6% and lPTS was 1%, respectively. Multivariate analysis revealed that both, history of brain operation or head injury and CT findings like traumatic focal HCs (in temporal lobe), were independent risk factors of lPTS. lPTS was higher in Group A when compared with Group B (p < 0.05), thus early treatment with AED seems to not affect the incidence of lPTS. But an AED with a mean time of initiation of 7.5 days (i.e., at late stage) from the mTBI occurrence could reduce the lPTS incidence. Future studies including MRI scans as a screening procedure into the emergency departments could be beneficial to determine the existence of traumatic lesions, that cannot be visible using only CT scan.

## Future perspective

Future studies including MRI scan as a screening procedure into the emergency departments could be beneficial to determine the existence of traumatic lesions, that cannot be visible using only a CT scan. Our study may be used to measure patients’ response to AED and may improve their outcomes. New methods are being developed to quantify injure on images and perhaps improve predictive power.

**Executive summary**

- We believe that an association between the late post-traumatic seizures (lPTS) and anti-epileptic drug (AED) exists. Both history of brain operation or traumatic brain injury and computer tomography findings such as traumatic focal hemorrhagic contusions (temporal), were independent risk factors of lPTS.
- Early treatment with AED seems to not affect the incidence of lPTS.
- AED with a mean time of initiation of 7.5 days from the moderate traumatic brain injury occurrence could reduce the lPTS incidence.
